# Evolution to permanent or transient conditions in children with positive neonatal TSH screening tests in Sergipe, Brazil

**DOI:** 10.1590/2359-3997000000189

**Published:** 2016-08-23

**Authors:** Diana M. Matos, Roberto J. R. Ramalho, Bruno M. Carvalho, Maria Augusta C. T. Almeida, Luciana F. D. Passos, Talmay T. S. Vasconcelos, Enaldo V. Melo, Carla R. P. Oliveira, Elenilde G. Santos, Karla F. Resende, Manuel H. Aguiar-Oliveira

**Affiliations:** 1 Departamento de Medicina Hospital Universitário Universidade Federal de Sergipe Aracaju SE Brasil Departamento de Medicina, Hospital Universitário, Universidade Federal de Sergipe (UFS), Aracaju, SE, Brasil

**Keywords:** Neonatal screening, congenital hypothyroidism, thyrotropin, thyroid hormones

## Abstract

**Objectives:**

To assess the evolution to permanent or transient conditions in children with positive neonatal TSH tests in Sergipe, Brazil, from 2004 to 2010.

**Subjects and methods:**

Out of 193,794 screened newborns, 713 presented a neonatal TSH level higher than the local cutoff (5.2 µU/mL). From the confirmatory serum TSH values, the children were diagnosed with initial congenital hypothyroidism (CH) or suspect CH. From the evolution, they were classified as permanent CH, hyperthyrotropinemia, or transient TSH elevation. The mean incidence of each final condition was calculated for the total period of time.

**Results:**

The initial diagnosis included 37 CH (18.1%) and 167 suspect CH (81.9%) cases. The final diagnosis included 46 cases of permanent CH (22.5%), 56 of hyperthyrotropinemia (27.5%), and 102 of transient TSH elevation (50.0%). Out of the 37 cases of initial CH, 23 (62.2%) had permanent CH, nine (24.3%) had hyperthyrotropinemia, and five (13.5%) had transient TSH elevation. Out of the 167 suspect CH cases, 23 (13.8%) had permanent CH, 47 (28.1%) had hyperthyrotropinemia and 97 (58.1%) had transient TSH elevation. The mean incidence after the follow up was 1:4,166 for permanent CH, 1:3,448 for hyperthyrotropinemia, and 1:1,887 for transient TSH elevation. Eighty-six percent of the children with an initial diagnosis of CH and 41.9% with suspect CH had a permanent condition (CH or hyperthyrotropinemia).

**Conclusions:**

The follow-up of children with an initial diagnosis of CH or suspect CH is necessary to determine whether the disorder is permanent because predicting the evolution of the condition is difficult.

## INTRODUCTION

Hypothyroidism is a clinical condition resulting from a decreased production or inadequate action of thyroid hormones (TH). Congenital hypothyroidism (CH) is often primary and diagnosed by an increase in serum thyroid stimulating hormone (TSH) associated with reduced thyroxine (T4). CH may be permanent or transient. Permanent CH results from the abnormal development of the thyroid gland (dysgenesis) or from a failure in hormone production by the thyroid (dyshormonogenesis). Transient CH, defined as remission before introduction or after withdrawal of l-thyroxine, is more common in premature newborns and is mostly caused by the transplacental passage of maternal TSH receptor blocking antibodies, maternal exposure to antithyroid drugs, iodine deficiency or excess (1,2).

Hyperthyrotropinemia is defined by high levels of TSH with normal T4 and, during the neonatal period, is caused mainly by the late maturation of the hypothalamic-pituitary axis, the presence of antithyroid antibodies, changes in thyroid morphology, and mild defects of thyroperoxidase or TSH receptor genes (3). While transient CH often remits within 4 to 6 weeks of life, the distinction between transient and permanent hyperthyrotropinemia generally requires a longer observation (2). When the distinction between transient CH and transient hyperthyrotropinemia is not possible, the use of the term “transient TSH elevation” is preferable. In this study, we defined hyperthyrotropinemia as a permanent condition (persistent hyperthyrotropinemia).

Treatment of transient CH and hyperthyrotropinemia is still controversial (1), but some authors have reported that it may have beneficial effects on full brain function of the affected children (4,5). If these children are treated, a definitive diagnosis should be sought after three years of age (2).

Before neonatal screening programs (NSP), the incidence of CH was estimated to be between 1:7,000 and 1:10,000 (6). With NSP, the incidence in the United States rose from 1:3,985 in 1987 to 1:2,273 in 2002 (7). However, there is wide variation in the incidence of CH worldwide. In Europe, a mean incidence of 1:2,709 has been reported, ranging from 1:1,333 in Greece to 1:13,886 in Estonia (8). Several factors may influence the incidence of CH including ethnicity, environmental factors, prematurity, the TSH cutoff points, and the different concepts and methodologies adopted by the programs (2,9-11).

The Brazilian Health Ministry Policy established in 2001 the University Hospital of the Federal University of Sergipe (HU-UFS) as the unique entity to perform the NSP in the State of Sergipe, which is mandatory in the public health care system (11,12). Since 2004, the cutoff value of TSH used in the Neonatal Screening Program in Sergipe (NSP-SE) is 5.2 µU/mL (11), which is less than the suggested 10 µU/mL in the manual of technical standards and operational routines of the National Neonatal Screening Program (NNSP) (13) but in line with a global trend to reduce the neonatal cutoff values (14).

Every year, hundreds of millions of CH screenings are performed around the world. With neonatal TSH cutoff reduction (less than 10 µU/mL), the false-positive rate has doubled, creating anxiety for families and program costs. The evolution to permanent and transient CH is controversial, and data on the evolution of these children are needed (15).

The objectives of this study were to assess the evolution to permanent or transient conditions in children with positive neonatal TSH screening tests in NSP-SE and to estimate the mean incidence of permanent CH, hyperthyrotropinemia and transient TSH elevation from 2004 to 2010 in the state of Sergipe.

## SUBJECTS AND METHODS

An analytical retrospective study was performed based on data from NSP-SE children with a positive neonatal TSH screening test from January 2004 to December 2010. The inclusion criteria were neonatal TSH more than 5.2 µU/mL (11) and confirmatory serum TSH more than 4.2 µU/mL. The exclusion criteria were the absence of files at the University Hospital outpatient facility and loss of follow-up before establishing a final diagnosis. The normal reference values of our laboratory were serum TSH less than or equal to 4.2 µU/mL, free T4 greater than or equal to 0.79 ng/dL, and total T4 greater than or equal to 7.2 µg/dL. For the initial diagnosis, we used only the TSH values. For the final diagnosis, we used TSH and free or total T4.

The children were classified according to the newborn TSH levels measured by fluoroimmunoassay Auto-DELFIA (*Perkin-Elmer Life Sciences, Turku, Finland*) as:

- Normal: neonatal TSH less than or equal to 5.2 µU/mL;
- Positive neonatal TSH: values greater than 5.2 µU/mL.

All of the serum confirmatory TSH assays were performed in the same laboratory (fluoroimmunoassay). The intra-assay and inter-assay variation coefficients were 5.2% and 8.4%, respectively. According to the serum TSH, the children were initially classified as:

- Normal (false-positive): serum TSH level lower or equal to 4.2 µU/mL;
- Initial CH: serum TSH level greater than 10 µU/mL;
- Suspect CH: serum TSH level greater than 4.2 µU/mL and less than or equal to 10 µU/mL.

The criteria for the final diagnosis were:

- Permanent CH: serum TSH level greater than 10 µU/mL, independent of the T4 levels or current use of thyroxine; or without thyroxine use, a serum TSH level between 4.2 µU/mL and 10 µU/mL, but with low free and/or total T4;
- Hyperthyrotropinemia: children without thyroxine use with a serum TSH level between 4.2 µU/mL and 10 µU/mL, with normal free T4 and total T4;
- Transient TSH elevation: initial CH or suspect CH children, which normalized in the follow up without thyroxine treatment (serum TSH level less than or equal to 4.2 µU/mL, free T4 greater than or equal to 0.79 ng/dL, and total T4 greater than or equal to 7.2 µg/dL). We used the term transient TSH elevation instead of transient CH because we included in this category both initial CH and suspect CH children that normalized.

**Figure 1:**
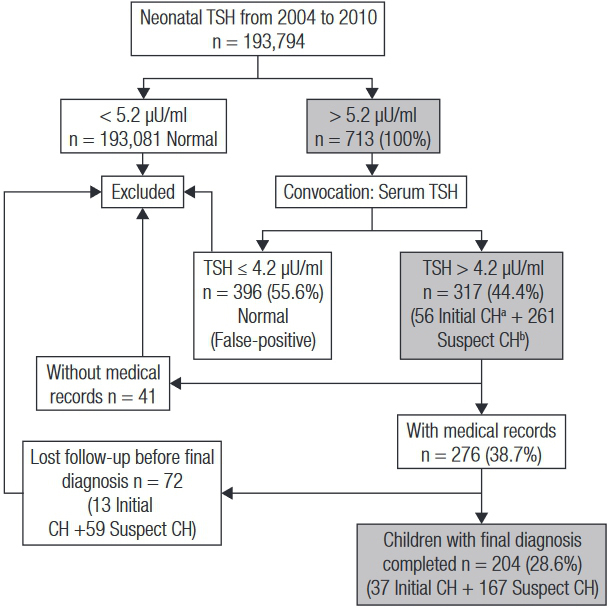
Flow chart of the sample selection of children screened by NSP-SE from 2004 to 2010, which included 204 children for the analysis.

^a^ TSH > 10 µU/ml.

^b^ TSH > 4.2 µU/ml and ≤ 10 µU/ml.

Out of 251,817 newborns from 2004 to 2010, 193,794 newborns were screened by NSP-SE (76.96%); 713 exhibited a positive neonatal TSH test (0.368%). Out of these, 317 (44.5%) presented with increased confirmatory serum TSH levels. Their records were sought in the outpatient clinic of the HU-UFS to evaluate the follow-up and final diagnosis. The children whose medical records were missing or lost follow-up before completing the final diagnosis were excluded from the study. Two hundred and four children were eligible (28.6% of positive tests), with their initial and final diagnosis (Figure 1). Most of the children were born at term (84.8%) and were male (56.9%). Out of 204 children, eight were preterm (two with permanent CH, two with transient TSH and four with hyperthyrotropinemia). The IRB Committee of the Federal University of Sergipe approved the research project.

Statistical analysis was performed using IBM SPSS (Statistical Package for the Social Sciences), version 21.0. TSH, and the ages of children at the events of NSP-SE were expressed as the median (interquartile range) without normal distribution. The minimum and maximum values are also shown. The categorical variables are summarized as simple frequency and percentage. The mean incidence of each final condition, after the following, was calculated by WinPepi version 4.0 by dividing the number of affected children with one of the three conditions for the number of children screened during the period. We did not know the number of initial CH from the private service to calculate the true incidence in Sergipe. The estimated sensitivity and specificity was calculated by the percentage and confidence interval (CI). The accepted level of significance was less than 0.05.

## RESULTS

Out of the 204 children, the initial CH diagnosis accounted for 18.1% (n = 37) and suspect CH accounted for 81.9% (n = 167). At the time of the final diagnosis, the permanent CH rate was 22.5% (n = 46), hyperthyrotropinemia was 27.5% (n = 56), and transient TSH elevation was 50.0% (n = 102). Permanent conditions together (permanent CH and hyperthyrotropinemia) corresponded to half of the children, and the other half corresponded to transient TSH elevation.

**Figure 2:**
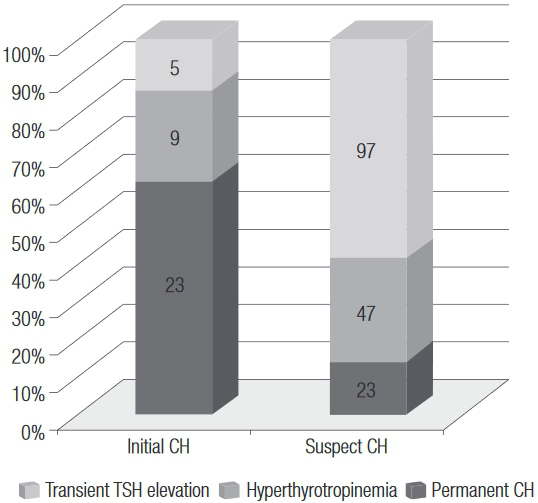
Final diagnoses of 37 initial CH and 167 suspect CH cases in 204 children with a completed final diagnosis screened by NSP-SE from 2004 to 2010.

Out of 37 children diagnosed with initial CH, on follow up, 23 (62.2%) had permanent CH, 9 (24.3%) progressed as hyperthyrotropinemia, and five (13.5%) had only transient TSH elevation. Among 167 children with a suspect CH initial diagnosis, 23 (13.8%) had a final diagnosis as permanent CH, 47 (28.1%) were diagnosed with hyperthyrotropinemia, and 97 (58.1%) had transient TSH elevation (Figure 2). The frequency of permanent CH was higher in the group with initial CH than in that with suspect CH (p < 0.001), but 23 children in the latter group had permanent CH.

The median (interquartile range) of neonatal TSH was 6.51 (2.58) µU/mL, ranging from 5.24 to 230 µU/mL. In our sample, 172 children had neonatal TSH between 5.21 and 10 µU/mL. Among them, transient TSH elevation had a higher frequency than hyperthyrotropinemia or permanent CH (54.7%, 29.6% and 15.7%, respectively). Thirty-two children had a neonatal TSH level above 10 µU/mL. Among these children, permanent CH was present in a higher frequency than transient TSH elevation or hyperthyrotropinemia (59.4%, 25.0% and 15.6%, respectively), (Table 1). The frequency of permanent CH was higher in the group with an initial neonatal TSH above 10 µU/mL than between 5.21 and 10 µU/mL (p < 0.001), but 27 children in the last group had permanent CH (Table 1).

**Table 1:** Final diagnosis for the neonatal TSH range of 204 children screened by NSP-SE from 2004 to 2010

Final diagnosis	Neonatal TSH 5.21 a 10.00 µU/mL n (%)	Neonatal TSH > 10.00 µU/mL n (%)
Permanent CH	27 (15.7)	19 (59.4)
Hyperthyrotropinemia	51 (29.6)	5 (15.6)
Transient TSH elevation	94 (54.7)	8 (25.0)
Total	172 (100.0)	32 (100.0)

The median (interquartile range) of confirmatory serum TSH was 5.85 (3.38) µU/mL, ranging from 4.21 to 743 µU/mL. In our sample, 167 children had a serum confirmatory TSH level between 4.21 and 10 µU/mL. Among these children, the transient TSH elevation had a higher frequency than hyperthyrotropinemia or permanent CH (58.1%, 28.1% and 13.8%, respectively). Thirty-seven children had a serum confirmatory TSH level above 10 µU/mL. Among these children, permanent CH presented with a higher frequency than hyperthyrotropinemia or transient TSH elevation (62.2%, 24.3% and 13.5%, respectively), (Table 2).

**Table 2:** Final diagnosis for two confirmatory serum TSH ranges of 204 children screened by NSP-SE from 2004 to 2010

Final diagnosis	Serum TSH 4.21 a 10.00 µU/mL n (%)	Serum TSH > 10.00 µU/mL n (%)
Permanent CH	23 (13.8)	23 (62.2)
Hyperthyrotropinemia	47 (28.1)	9 (24.3)
Transient TSH elevation	97 (58.1)	5 (13.5)
Total	167 (100.0)	37 (100.0)

In Table 3, we correlate the final diagnosis with the first serum TSH range (confirmatory TSH), subdividing it into two groups which was: between 4.21 and 6 µU/mL and between 6.01 and 10 µU/mL. We found that seven children (6.6%) who had permanent CH in the evolution presented the first serum TSH between 4.21 and 6 µU/mL, corresponding to 15.2% of permanent CH cases.

**Table 3:** Final diagnosis for three confirmatory serum TSH ranges of 204 children screened by NSP-SE from 2004 to 2010

Final diagnoses	Serum TSH 4.21 a 6.00 µU/mL n (%)	Serum TSH 6.01 a 10.00 µU/mL n (%)	Serum TSH > 10.00 µU/mL n (%)
Permanent CH	7 (6.6)	16 (26.2)	23 (62.2)
Hyperthyrotropinemia	30 (28.3)	17 (27.9)	9 (24.3)
Transient TSH elevation	69 (65.1)	28 (45.9)	5 (13.5)
Total	106 (100.0)	61 (100.0)	37 (100.0)

Table 4 shows the initial categories (initial CH or suspect CH) corresponding to permanent condition diagnoses (CH and hyperthyrotropinemia or transient TSH elevation). The estimated sensitivity was 84.21% (CI 69.58% to 92.56%) and specificity was 57.83% (CI 50.23% to 65.08%).

**Table 4:** Initial classification and final diagnosis of 204 children screened by NSP-SE from 2004 to 2010

Initial classification	Permanent condition (CH or hyperthyrotropinemia)	Transient TSH elevation	Total
Initial CH	32	5	37
Suspect CH	70	97	167
Total	102	102	204

The sensitivity was 84.21% and specificity was 57.83%.

The mean time of follow-up was 1072 days. Permanent conditions together (permanent CH and hyperthyrotropinemia) had a mean time of follow-up of 1571 days. Permanent CH had a mean time of follow-up of 2030 days, hyperthyrotropinemia of 1195 days and transient TSH elevation of 584 days.

The median (interquartile range) time of neonatal TSH collection was 5 (4) days of life, arrival to the laboratory was 8 (9) days, obtaining the results of neonatal TSH was 11 (13) days, the serum confirmatory TSH collection was 48 (25) days of life and the first consultation was 60 (34) days of life (minimum of 22 and maximum of 257).

From 2004 to 2010, the mean incidence of initial CH was 1:3,461 and the final diagnosis was 1:4,166 for permanent CH; 1:3,448 for hyperthyrotropinemia; and 1:1,887 for transient TSH elevation.

## DISCUSSION

In this neonatal screening program, the follow-up of children with an abnormal confirmatory TSH test revealed that half had a final diagnosis of permanent condition (22.5% of permanent CH and 27.5% of hyperthyrotropinemia). Most of the children with an initial diagnosis of CH (86.5%) and 41.9% with suspect CH have a permanent condition (CH or hyperthyrotropinemia). We observed a higher frequency of progression to permanent CH in children with an initial CH diagnosis and a higher frequency of transient TSH elevation in children with an initial suspect CH diagnosis. Nevertheless, 13.8% of the suspect CH children had permanent CH, thereby justifying the follow-up of all children with an abnormal test.

Our rate of evolution to a permanent condition (86.5%) in the children diagnosed with initial CH is similar to that of the state of Michigan (USA), in which 75% of the diagnosed children progressed to permanent CH (16). In Paris, an analysis of 79 children with milder cases of CH and in-situ thyroid with less strict diagnostic criteria than ours (transient CH, TSH less than 6 µU/mL after cessation of treatment for at least 4 weeks and permanent CH, TSH greater than 7 µU/mL) revealed permanent CH in 62% and transient CH in 38% of the children (17). Another study in the Italian Lombardy region examined the follow-up of 84 children with CH and in-situ thyroid after the suspension of l-thyroxine after three years, and found that 34.5% of the children had permanent CH, 27.4% had hyperthyrotropinemia (TSH 5 to 10 µU/mL) and 38.1% had transient CH (18). Thus, the follow-up of children is necessary to characterize a permanent disorder, considering the suboptimal specificity of the screening test. Therefore, the prediction of the evolution of these children is difficult, even for those with in-situ thyroid.

During recent years, the concern for false-negative screening has been raised in Brazilian NSP, with different neonatal TSH cutoffs (19,20). In our sample, over half of the children with permanent CH and most of the hyperthyrotropinemia cases were diagnosed with neonatal TSH between 5.2 and 10 µU/mL. A neonatal TSH cutoff of 10 µU/mL would have missed 78 cases that resulted in a permanent condition (27 cases of CH and 51 of hyperthyrotropinemia). In Greece, with the reduction of the neonatal TSH cutoff from 20 to 10 µU/mL, there was an increase of 28% in the number of treated children but a tenfold increase of false-positive rates; however, the majority of children (88.7%) showed permanent CH, indicating that the increased incidence of CH after reducing the cutoff point was not due to transient TSH elevation (21). On the other hand, when the serum confirmatory TSH was analyzed, we found that half of the children with permanent CH and most with hyperthyrotropinemia were diagnosed with confirmatory serum TSH levels between 4.2 and 10 µU/mL. The cutoff of confirmatory serum TSH of 10 µU/mL would imply a loss of 70 cases of permanent conditions (23 CH and 47 hyperthyrotropinemia). We also found that 15.2% of the children with serum TSH levels between 4.2 and 6 µU/mL would not have been diagnosed if the TSH cutoff point was 6 µU/mL, according to the national recommendation (22).

Corbetta and cols., 2009, decreased the neonatal TSH cutoff from 20 µU/mL to 12 µU/mL (1999 to 2002) and 10 µU/mL (2003 to 2005) and observed that 45% of the children with CH would have been missed with the previous cutoff, and 78% had permanent thyroid dysfunction (14). Korada and cols., 2010, decreased the limit for the TSH cutoff from 20 µU/mL to 6 µU/mL and observed that, in 67 children with TSH between 6.1 and 10 µU/mL, 4 continued to display TSH levels greater than 6 µU/mL (23). In the Brazilian state of Santa Catarina, the recommended cutoff point is 6 µU/mL because, at 10 µU/mL, some CH cases can be missed (20). A study conducted in Rio de Janeiro, Brazil, found a significant number of cases of permanent CH screened with neonatal TSH levels between 4.5 and 10 µU/mL (62.6% with confirmatory TSH > 10 µU/mL), recommending a neonatal TSH cutoff value of 4.5 µU/mL to prevent missed cases despite the inevitable necessity of more confirmatory tests (24).

Thyroid hormones are essential for the normal maturation of neurons, and the second and third weeks of life are crucial for the treatment of hypothyroidism to minimize the time when the brain is exposed to hypothyroxinemia, prevent severe neurological damage, and to result in in normal or near normal functioning in children with CH (14,15,25). In our program, the median age at the time of neonatal TSH collection was five days, later than the recommended ideal time of up to four days (1,13). Thus, because the concentration of neonatal TSH decreases with age until it stabilizes at between 11 and 15 days of life, the cutoff point should be adapted to the reality of time collection in each population (11). Altitude (Sergipe does not have high mountains) and the hot weather in Sergipe [mean annual temperature between 25 and 26°C(26)] seem not to significantly reduce the neonatal TSH measurements in this Brazilian northeastern state (unpublished data); however, delayed collection and measurement issues are not the only reasons for a reduction in the neonatal TSH cutoff. Ethnic factors, as discussed later, seem to be the most plausible reason for this global trend of reduction, independent of the collection and testing time or cutoff levels (27).

A secondary purpose of this work was to establish the mean incidence of each of the final diagnoses during the seventh year of this study. The mean incidence of permanent CH was 1:4,166, hyperthyrotropinemia was 1:3,448, and transient TSH elevation was 1:1,887. A group in Italy found an incidence of 1:1,446 of confirmed CH cases using a cutoff of 10 to 12 µU/mL between 1999 and 2005 (14). In addition to the global trend to reduce the cutoff point in neonatal screening, thereby including hyperthyrotropinemia and transient TSH elevation, another factor related to the higher incidence of CH is ethnic composition. Hispanics and Asians are reported to have the highest CH incidences (1:1,600 and 1:2,380, respectively) (9). Sixty percent of the population of northeastern Brazil is of European origin (28), and Sergipe is one of the regions where the Portuguese began the colonization of Brazil. The Iberian descent may have caused an increased incidence of CH. Accordingly, the lower neonatal TSH cutoff by six NBS programs around the word, when TSH cutoff was lowered from a range of 20-25 µU/mL to 6-10 µU/mL (whole blood), reportedly doubled the incidence of CH, increasing from 1:3,500 to 1:1,714 (9).

The analysis of the chronology of events in our NSP reveals a longer than ideal time lapse between the results of neonatal TSH analysis and collection of the confirmatory test (the median was 48 days old, as previously reported) (11). An increase in the speed of the recall process to bring our median age of diagnosis (presently 60 days) to the recommended age of 14 days of life is imperative (1,13).

All of 317 children with an abnormal confirmatory TSH value should have been enrolled in the outpatient follow-up, but 12.9% of them did not have identified file records. In addition, the rate of follow-up loss before completing the final diagnosis was 22.6%. If we take into account loss of follow-up in general, including cases with a completed final diagnosis, this figure rises to 38.0%, similar to two different reports (44.7% and 38.0%) from the USA. These problems are linked to financial constraints and other barriers (16,29). In addition, few screening programs follow-up routinely detected cases beyond the diagnosis. Such data demonstrate the need to monitor the follow-up of children as performed in diseases with compulsory notification.

The limitations of our study include the quality of the file data and the difficulty of comparison with other studies with different methodologies, concepts (CH, hyperthyrotropinemia and transient TSH elevation), and cutoff values of TSH for both the screening and confirmation of CH.

In conclusion, children with an initial diagnosis of CH evolve mostly to permanent conditions, while suspect CH cases are more likely to be diagnosed with transient TSH elevation. Because it is difficult to predict which of the positive cases will evolve to a permanent abnormality, close monitoring of all the cases with an initial diagnosis of CH or suspect CH is mandatory.
